# Comparative study of the gut microbiome potentially related to milk protein in Murrah buffaloes (Bubalus bubalis) and Chinese Holstein cattle

**DOI:** 10.1038/srep42189

**Published:** 2017-02-08

**Authors:** Jiachao Zhang, Chuanbiao Xu, Dongxue Huo, Qisong Hu, Qiannan Peng

**Affiliations:** 1 College of Food Science and Technology, Hainan University, Haikou 570228, P. R. China

## Abstract

Previous studies suggested a close relationship between ruminant gut microbes and the mammary gland. In this study, shotgun metagenomic sequencing was used to reveal the differences in the intestinal microbiome potentially related to milk components in Murrah buffaloes and Chinese Holstein cattle. A PCoA based on the weighted Unifrac distances showed an apparent clustering pattern in the structure of intestinal microbiota between buffalo and cattle. We could attribute the structural difference to the genera of *Sutterella, Coprococcus* and *Dorea*. A further analysis of microbial functional features revealed that the biosynthesis of amino acids (including lysine, valine, leucine and isoleucine), lipopolysaccharide biosynthesis and cofactor/vitamin biosynthesis were enriched in the buffalo. In contrast, dairy cattle had higher levels of pyruvate metabolism and carbon fixation in photosynthetic organisms. A further correlation analysis based on different milk components and the typical microbiome uncovered a significant positive correlation between milk protein and the microbial biosynthesis of amino acids, which was also positively correlated in the genera of *Parabacteroides, Dorea* and *Sutterella*. This study will expand our understanding of the intestinal microbiome of buffalo and cattle as representative ruminants, as well as provide new views about how to improve the production and nutritional qualities of animal milk.

Murrah buffaloes (Bubalus bubalis) and Chinese Holstein cattle, which belong to the genus *Bos* (order: *Artiodactyla;* suborder: *Ruminantia)*, are economically significant livestock that have been used as a source of dairy and meat, as well as draught power^1^. Due to their biological characteristics and the natural resources in China, dairy cattle and native swamp buffalo are mainly distributed in northern and southern China, respectively^2^,^3^,^4^. Compared to small poultry livestock, buffalo and dairy cattle have the advantage of high roughage feeding, anti-disease and anti-adversity, high reproductive rates and hereditary stability. Additionally, the milk is rich in various nutrients, including protein, fat, vitamins, and minerals. Milk is therefore considered a perfect food with a relatively complete nutritional structure^5^.

The animal gastrointestinal tract is a major habitat for numerous species of microbes. This cohort consists of at least 10^14^ members and is dominated by anaerobic bacteria^6^. Gut microbiota play important roles in extracting nutrients from the host diet, regulating host fat storage, stimulating intestinal epithelium renewal and directing maturation of the immune system^7^. In addition, previous studies suggested a close relationship between ruminant gut microbes and the mammary gland. Jimenez^8^ reported the oral administration of *Lactobacillus* strains isolated from breast milk as an alternative for the treatment of infectious mastitis during lactation. Another earlier study also clarified the potential mechanism between the occurrence/development of mastitis and intestinal microbiota^9^. The mammary gland is the only site of milk production in animals, so we hypothesized that the intestinal microbiota was able to influence the composition and nutrition of animal milk by acting on the mammary gland.

With the development of next-generation sequencing, we described the microbial diversity in any micro-ecosystem globally by high-throughput sequencing of bacterial 16S rRNA. Furthermore, the improvements in shotgun metagenomic sequencing allow us to explore the microbial interaction and functional features at the metabolic level. By combining the pyrosequencing of the metagenomic DNA and fibrolytic active BAC clones prepared with the same DNA pool, Xin Dai^10^ revealed the profile of the fibrolytic genes that are indicative of lignocellulose degradation mechanisms in the yak rumen. To characterize biomass-degrading relative functional genes, Hess *et al*.^11^ explored deeply sequenced metagenomic reads (268 G bases) from complex microbiota related to plant fibre incubated in cow rumen. From the results, they identified 27,755 carbohydrate-active genes and over 90 candidate proteins, of which more than 57% were enzymatically active against cellulosic substrates. To explore the relationship between bacterial taxa of the human gut microbiota and those in the gut microbiota of domestic and semi-wild animals, Ellis^12^ compared the distal gut microbiota of humans, cattle and semi-captive chimpanzees in communities that are geographically sympatric in Uganda. The results indicated a unique intestinal microbial profile in cattle, which characterized by abundant of the genus *Ruminococcus* and the high level of the ratio of Firmicutes and Bacteroidetes. Meanwhile, the genera *Anoxybacillus, Clostridium* and *Enterobacteriaceae* were identified closely related to the multiple carbohydrate fermentation.

China is the major milk producing country in Asia, and dairy cattle and buffalo are the most important cow species in the northern and southern regions of China. Based on a previous correlation study between the cow mammary gland and gut microbes^13^, we designed a study to investigate the differences in typical intestinal microbiomes and its potential correlation with milk components by shotgun metagenomic sequencing. This study will supply the theoretical foundation for further use of probiotics to modulate the balance of host intestinal microbiota. Moreover, we also provide a new view for increasing milk production and quality, as well as promoting the long-term development of China’s dairy industry.

## Results

### Sequencing coverage and estimation of bacterial alpha diversity

In this study, shotgun metagenomic sequencing and 16S rRNA high throughput sequencing were applied to reveal the differences in the intestinal microbiome between buffalo (n = 10) and dairy cattle (n = 10). The structure of intestinal microbiota was analysed by 16S rRNA gene sequencing reads, which revealed for each microbiota on average 2,165 operational taxonomic units (OTUs) from an average of 8,173 reads (Table 1). To characterize the functional profiles of the microbiota, all samples were selected for whole-metagenome shotgun sequencing, yielding 197.4 Giga base (Gb) of pair-end reads (averagely 61,837,672 high-quality reads for each microbiota; Table 1). By determining the alpha diversity within samples, we observed no significant difference in microbes between buffalo and cattle (Table 1).

### The intestinal microbial diversity in buffalo and cattle

**Figure 1:**
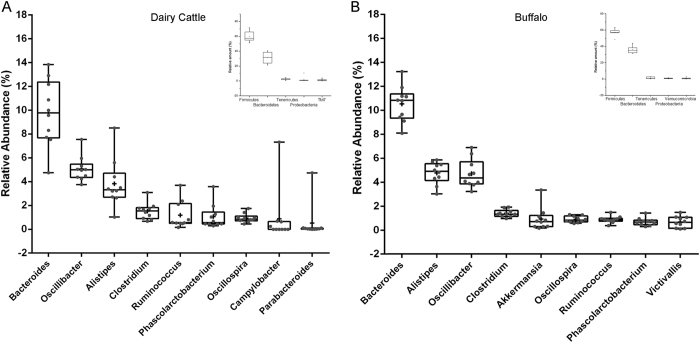
The composition of intestinal microbiota of dairy cattle (**A**) and buffalo (**B**) at the phylum and genus level.

At the phylum level, the Firmicutes, Bacteroidetes, Tenericutes and Proteobacteria (contributing to 95.38% of the total number of sequences) dominated the intestinal microbiota in both buffalo and dairy cattle. At the genus level, *Bacteroides* was the most abundant genus (contributing to 9.77% of the total number of sequences) in dairy cattle, and the amounts of *Oscillibacter, Alistipes, Clostridium, Ruminococcus* and *Phascolarctobacterium* all exceeded 1% (Fig. 1A). In buffalo, the *Bacteroides* was also the predominant genus, followed by *Alistipes, Oscillibacter, Clostridium, Akkermansia* and *Oscillospira* (Fig. 1B). Thus, the predominant genera in buffalo and cattle were similar, but the amounts of these genera were different.

### Differences in gut microbiota between buffalo and cattle

**Figure 2:**
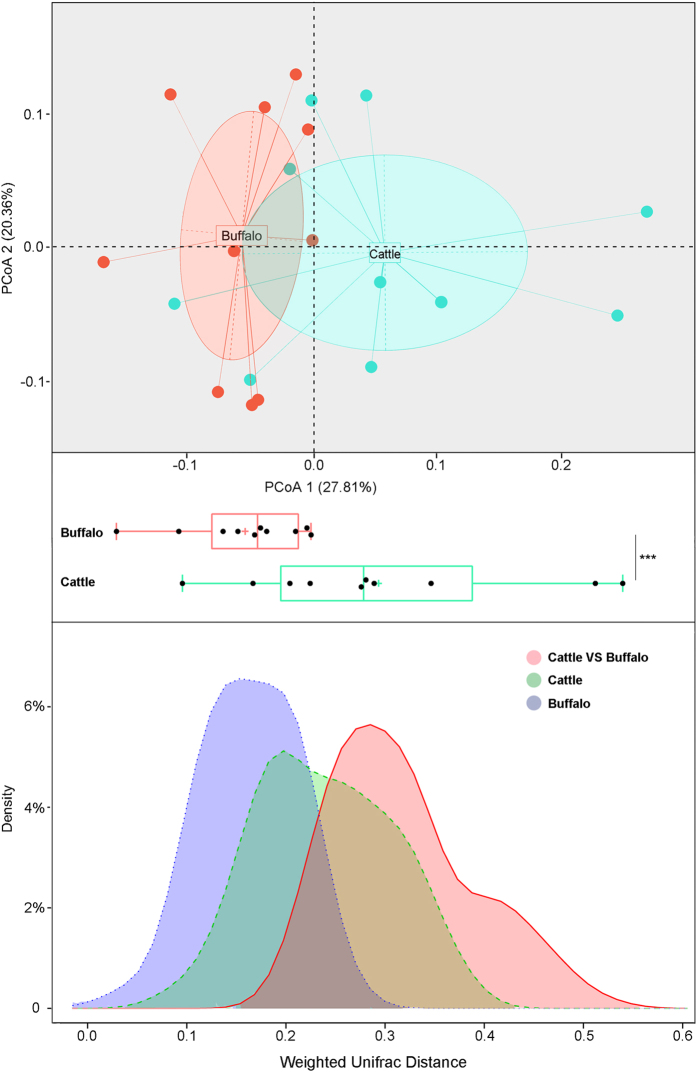
Differences in gut microbiota between buffalo and cattle. (**A**) A principal component (PCoA) score plot based on weighted UniFrac metrics for all samples. Each point represents the composition of the intestinal microbiota of one sample. (**B**) The kernel density profile based on weighted UniFrac distance within and between buffalo and cattle.

To test whether any differences in the organismal structure of intestinal microbiota were present, a principal coordinates analysis (PCoA) was performed based on the weighted Unifrac distances of 16S rRNA sequence profiles at the OTUs level. As shown in Fig. 2A (upper panel), an apparent clustering pattern was identified for the blue and red points, which represent intestinal samples from cattle and buffalo, respectively, and significant separation (*P* < 0.001) in PC 1 was observed (Fig. 2A, lower panel; Wilcoxon rank-sum tests). This result was confirmed by the kernel density distribution profile of weighted Unifrac distances between buffalo and cattle (Fig. 2B).

**Figure 3:**
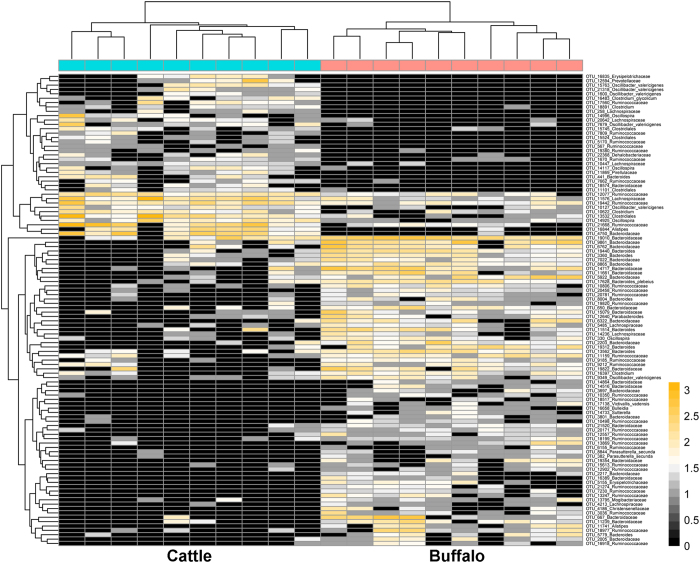
Heatmap constructed using the amount of significantly different OTUs between buffalo and cattle samples by the Wilcoxon rank-sum test.

After confirming that there was an intrinsic difference in intestinal microbiota composition between buffalo and cattle, we further identified differences in specific bacteria at the genus and OTUs level (Table 2 and Fig. 3). At the genus level, the relative amount of *Sutterella, Coprococcus, Parasutterella, Paludibacter* and *Dorea* were significantly higher in the buffalo group, whereas *Streptococcus, Pseudobutyrivibrio, Anaerorhabdus, Campylobacter* and *Blautia* were enriched in the cattle group. A further analysis according to the OTUs profile revealed a total of 108 OTUs that were significantly different between buffalo and cattle. Among these OTUs, 70 OTUs mainly belonged to the order of *Bacteroidaceae* and *Ruminococcaceae,* and the genus of *Bacteroides* was enriched in the buffalo group. Meanwhile, 38 OTUs, mainly belonging to the *Lachnospiraceae* order, *Clostridiales* genus and *Oscillibacter valericigenes,* were enriched in the cattle group.

### Typical microbial functional features and metabolic pathways identified from cattle and buffalo samples

**Figure 4:**
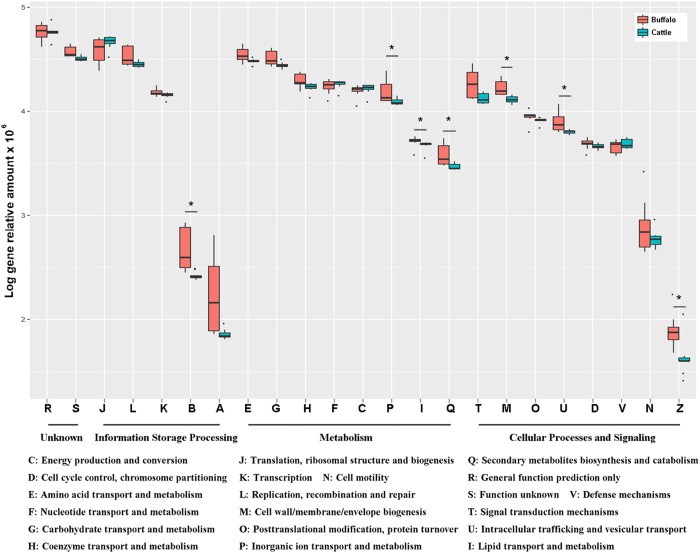
Comparison of the microbial functional features (COGs) between the buffalo and cattle groups.

To identify the intestinal microbial functional differences between buffalo and cattle, the shotgun metagenomic data were annotated using the COG and the KEGG databases, and analysed at the functional and metabolic pathway levels. The results revealed that 7,409 KOs and 1,917 COGs were identified either in buffalo or cattle samples. By comparing the profile of COGs functional features, we found that the COGs that were enriched in buffalo include those implicated in chromatin structure and dynamics; cell wall biogenesis; intracellular trafficking, secretion, and vesicular transport; amino acid transport and metabolism; lipid transport and metabolism; and inorganic ion transport and metabolism. In contrast, only the COGs of translation, ribosomal structure and biogenesis were enriched in the cattle group (Fig. 4).

**Figure 5:**
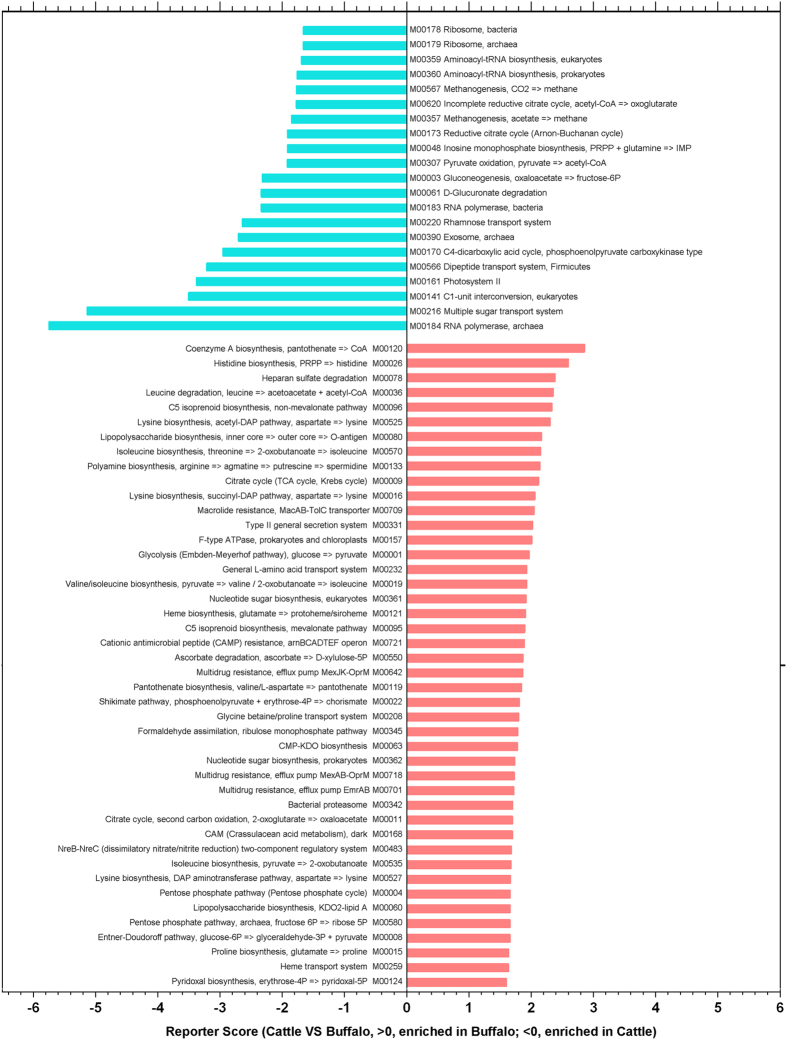
Microbial KO modules enriched in buffalo or cattle samples. The relative abundances of KO modules were compared between buffalo and cattle, and modules with a significant difference in reporter score (<−1.6, enriched in cattle; >1.6, enriched in buffalo) are shown.

We further mapped the KOs to KEGG modules and pathways, and calculated the reporter Z scores of each pathway or module by using the reporter feature algorithm (Table 3 and Fig. 5). A reporter score = 1.6 (90% confidence according to the normal distribution) was used as the detection threshold for significantly differentiating modules or pathways. In particular, the biosynthesis of amino acids (including lysine, valine, leucine and isoleucine), lipopolysaccharide biosynthesis, cofactor and vitamin biosynthesis, phosphotransferase system (PTS), peptidoglycan biosynthesis, biotin metabolism and amino sugar and nucleotide sugar metabolism were enriched in the buffalo. In contrast, the intestinal microbiome of dairy cattle had higher levels of KO modules which were involved in pyruvate metabolism, glycolysis/gluconeogenesis, nitrotoluene degradation and carbon fixation in photosynthetic organisms. Generally, the predominant metabolism in gut was introduced by anaerobic bacteria, and the metabolic pathway of carbon fixation in photosynthetic organisms was mainly introduced by the photosynthetic bacteria and the phylum Cyanobacteria. Given present research, we annotated abundant Cyanobacteria in distal gut samples (Table S2), and observed the amount of the phylum Cyanobacteria in cattle was higher than that in buffalo. Then we can attribute the high level of the metabolic pathway of carbon fixation in photosynthetic organisms in cattle to the more abundant of Cyanobacteria in gut.

### The determination of milk components and correlation with the intestinal microbiome

**Figure 6:**
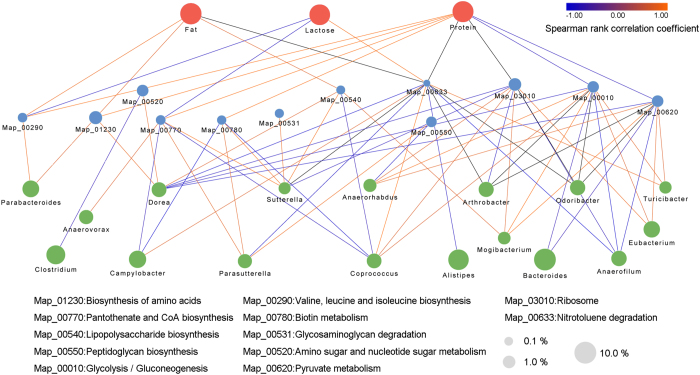
The correlation network of different milk components and typical metabolic pathways with relative genera. The edge width and colour (red: positive and blue: negative) are proportional to the correlation strength. The node size is proportional to the mean abundance in the respective population.

The common milk components, including protein, fat, lactose, total solids and major elements, between dairy cattle and buffalo were determined and compared (Table 4). The amounts of protein, fat, total solids and the elements Mg and Ca in the milk of buffalo were significantly higher than in dairy cattle. In contrast, lactose and vitamin K were enriched in cattle milk. To probe the potential mechanism underlying the milk differences, we determined the correlation among the different milk components, the typical microbial metabolic pathway and the relative genera according to Spearman’s rank correlation coefficient (R > 0.4, Fig. 6). The network revealed that the protein was positively correlated to the pathways of biosynthesis of amino acids, valine, leucine and isoleucine biosynthesis, pantothenate and COA biosynthesis and biotin metabolism, which also positively correlated to the genera of *Parabacteroides, Dorea, Sutterella* and *Parasutterella*. Meanwhile, the milk fat exhibited multiple and complex relationships with microbes and microbial pathways. On one hand, a negative correlation was observed between the milk fat content and the microbial pathway of nitrotoluene degradation, and the pathway was positively correlated to the genera *Campylobacter, Coprococcus* and *Turicibacter*, but negatively correlated to the genera *Dorea, Parasutterella, Alistipes* and *Anaerofilum*. On other hand, the positive correlations were found between the milk fat and the pathway of lipopolysaccharide biosynthesis, biosynthesis of amino acids, valine, leucine and isoleucine biosynthesis.

## Discussion

From the relative amount of major genera, we found that *Bacteroides, Oscillibacter, Alistipes, Ruminococcus* and *Clostridium* were the predominant bacteria in the gut of buffalo and cattle. The gastrointestinal tract of herbivores contains various microbes that harbour the complex lignocellulosic degradation system for the microbial attachment and digestion of plant biomass^14^. An example is *Bacteroides* spp, which digest a wide variety of otherwise indigestible dietary plant polysaccharides (e.g., amylose, amylopectin, and pullulan)^15^. In addition, *Alistipes*, a member of the family *Rickenellaceae*, might be a bacterial genus of particular interest in the field of fibre degradation^16^. In a previous study comparing the effects of plant- vs animal-derived diets on microbiota, *Alistipes* and *Bacteroides* were considered to be enriched in the former, plant-derived fibre-rich diet, consistent with a purported role in plant polysaccharide degradation^14^. The *Ruminococcus* are also considered to be the most important cellulose-degrading bacteria in the intestine of herbivores, and they produce large amounts of cellulolytic enzymes, including exoglucanases, endoglucanases, glucosidases and hemicellulases^17^. Accordingly, the high detection of *Alistipes, Bacteroides* and *Ruminococcus* in this study of intestinal microbiota in buffalo and cattle is associated with fibre degradation in feed. These bacteria also degraded xylan and pectin and utilize degraded soluble sugars as substrates.

To establish a unique catalogue of microbial reference genes is crucial for microbial functional metagenomic analyses. Although a large catalogue of reference genes from intestinal microbiota that included 9,879,896 genes was recently reported in humans^18^, it was mainly derived from healthy individuals from MetaHIT and HMP, which tended to limit its reference value for interpreting buffalo and cattle herbivorous microbiota. Additionally, numerous reports had dominated the significant difference in the composition and functional features of intestinal microbiota between human and herbivore. Thus, the intestinal gene catalogue established in this study of 3,172,144 microbial genes from both buffalo and cattle should be of value for future studies probing the role of the intestinal microbiome in herbivores.

To explore the potential correlation between milk components and the intestinal microbiome, a complex interactive network, including the common milk components, typical microbial pathways and matched genera, was established. A positive correlation was found between the protein amount and the pathways of biosynthesis of amino acids (including lysine, valine, leucine and isoleucine), which also positively correlated to the genera of *Parabacteroides, Dorea, Sutterella* and *Parasutterella*. Previous research had focused on the impact of gut microbes on the mammary gland and bovine mastitis. These studies described the drastic differences in both the milk and faecal microbial compositions in dairy cattle suffering mastitis and associated the structural changes with the loss of gut *Lactobacillus*^13^. There are two potential mechanisms for explaining the interaction between the gut microbes and the mammary gland. One possibility is that intestinal microbes are able to move through the dendritic cells in the intestinal epithelia by endocytosis and arrive in the mammary gland to play helpful or harmful roles^19^. Or alternatively, the microbial metabolite can move through the intestinal epithelial cells and enter the blood flow to the mammary gland, and then impact the milk components secreted by the mammary gland^20^. The second potential mechanism also highlights the importance of microbial function in our investigation.

In this study, a metagenomic approach was used to uncover the composition of intestinal microbiota and the microbial functional diversity in buffalo and cattle. A further correlation analysis uncovered the interaction between the intestinal microbiome and milk components. This research will expand our understanding of the intestinal microbiome of buffalo and cattle as representative ruminants and will also providing new ideas about how to improve the production and nutrition of animal milk.

## Materials and Methods

### Study design and sample collection

The cows studied in this research are Chinese Holstein cattle and the Murrah buffaloes (Bubalus bubalis). The research protocol was reviewed and approved by the Institutional Animal Care and Use Committee of Hainan University (Haikou, China). Fecal and milk samples of 5-year-old dairy cattle and buffalo were collected from two pastures in Haikou city and Danzhou city in Hainan province, respectively. Composition of the feeds for diets of cattle and buffaloes are the same using the commercial products.

The teat-ends were carefully disinfected before the milk samples were taken directly by hand in the morning. Faecal samples (10 g) were numbered (Cattle1–10, n = 10 and Buffalo1–10, n = 10), weighed, mixed with protector (Takara, Japan) in sterile tubes at the ratio of 1:5 (w/w) and vortex until homogenous. The samples were placed in an ice box for transportation to the laboratory, after which the metagenomic DNA was extracted immediately for subsequent bacterial 16S rRNA gene V3–V4 region high-throughput sequencing and metagenomic shotgun sequencing. The study was approved by the Ethical Committee of the Hainan University (Haikou, China). The materials and methods described in this research were conducted in accordance with the approved guidelines.

### Metagenomic DNA extraction

The QIAamp^®^ DNA Stool Mini Kit (Qiagen, Hilden, Germany) was used for DNA extraction from the faecal samples. The quality of the metagenomic DNA was assessed by 0.8% agarose gel electrophoresis. All of the DNA samples were stored at −20 °C until further processing.

### PCR amplification of the bacterial 16S rRNA gene V3–V4 region using high throughput sequencing and a bioinformatics analysis

The V3–V4 region of the 16S ribosomal RNA (rRNA) genes was amplified for each sample. A set of 8-nucleotide barcodes was added to the universal forward primer 338 F (5′-ACTCCTACGGGAGGCAGCA-3′) and the reverse primer 806 R (5′-GGACTACHVGGGTWTCTAAT-3′), which were targeted at the domain Bacteria. PCR amplification was then performed as described previously^21^. The PCR products were sequenced using the Illumina Miseq PE300 platform. High-quality sequences were extracted from the raw reads. After the removal of the barcodes and primer, the extracted sequences were processed mainly using the QIIME (v1.7.0) suite of software tools^22^.

### Shotgun metagenomic sequencing and quality control

The Illumina HiSeq2500 platform was used for shotgun metagenomic sequencing. Paired-end reads were generated with 100 bp in the forward and reverse directions. The length of each read was trimmed with Sickle. This set of high-quality reads was then used for a further analysis. An average of 9.87 gigabases (Gb) of paired-end reads were obtained for each sample, totalling 197.4 Gb of high-quality data free of host genomic and adaptor contaminants.

### Shotgun metagenomic reads, de novo assembly, gene prediction and construction of the non-redundant gene catalogue

The Illumina reads were assembled into contigs using IDBA-UD^23^ with default parameters. Genes were predicted on the contigs with MetaGeneMark^24^. A non-redundant gene catalogue was constructed with CD-HIT^25^ using a sequence identity cut-off of 0.95, with a minimum coverage cut-off of 0.9 for the shorter sequences. This catalogue contained 3,172,144 microbial genes.

### Annotation of COG and KEGG database

We aligned the amino acid sequences that were translated from the gene catalogue against the proteins/domains in the Cluster of Orthologous Groups (COG) and the Kyoto Encyclopedia of Genes and Genomes (KEGG) databases using BLASTP (e-value ≤ 1e-5 with a bit-score higher than 60). Each protein was assigned to the KEGG orthologue group (KO) or cluster of orthologous groups by the highest scoring annotated hit.

### Computation of relative gene abundance

To assess the abundance of genes profile, reads were aligned to the gene catalogue with Bowtie2^26^ using the following parameters: -p 12 -x nt -1 R1.fastq -2 R2.fastq -S R.sam. Then, for any sample N, we calculated the abundance as follows:

Step 1: Calculation of the copy number of each gene:


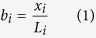


Step 2: Calculation of the relative abundance of gene i:


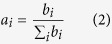


*a*_*i*_: the relative abundance of gene i

*b*_*i*_: the copy number of gene i from sample N

*L*_*i*_: the length of gene i

*x*_*i*_: the number of mapped reads

### KEGG pathway analysis

Differentially enriched KO modules and pathways were identified according to the reporter scores^27^ from the Z-scores of individual KOs. Accordingly, the Z _adjustedpathway_ of each KEGG module and pathway was calculated as previously described^27^. Then, the Z _adjustedpathway_ was used as the final reporter score for evaluating the enrichment of specific pathways or modules. A reporter score of >1.6 (90% confidence according to the normal distribution) could be used as a detection threshold for significantly differentiating pathways.

### The measurement of the milk components

The protein content was determined according to the Kjeldahl method (method 991.20; AOAC International, 2012),^28^ the lactose content was determined according to AOAC International (2012; method 896.01),^28^ and the total fat content was determined using the Mojonnier method according to AOAC International (2012; method 996.06)^28^. The percent ash used 2 g of sample in a crucible and subjected the sample to a high-temperature furnace at 550 °C for 60 min (method 930.30; AOAC International, 2012)^28^. Prior to the ashing procedures, milk samples were dried in an oven at 100 °C for 60 min to remove moisture and avoid splatter. The microelement composition including Na, Mg, K and Ca in milk was determined by inductively coupled plasma mass spectrometry (ICP-MS) according to Matos *et al*.^29^.

### Statistical analysis

All statistical analyses were undertaken using the R software. PCoA and a kernel density distribution analysis were performed in R using the ade4^30^ package. Differential abundance of genus, genes and COGs were tested by the Wilcoxon rank sum test. False discovery rate (FDR) values were estimated using the Benjamini-Yekutieli method^31^ to control for Wilcoxon rank sum test *P* value. The heatmap of significantly different OTUs was built in R using the “pheatmap” package. The correlation between the milk components, metabolic pathways and relative genera were calculated by Spearman’s rank correlation coefficient and visualized by network in Cytoscape (Version 3.2.1).

### Data availability

The sequence data reported in this paper have been deposited in the NCBI database (Metagenomic data: SRP075434).

## Additional Information

**How to cite this article:** Zhang, J. *et al*. Comparative study of the gut microbiome potentially related to milk protein in Murrah buffaloes (Bubalus bubalis) and Chinese Holstein cattle. *Sci. Rep.* **7**, 42189; doi: 10.1038/srep42189 (2017).

**Publisher's note:** Springer Nature remains neutral with regard to jurisdictional claims in published maps and institutional affiliations.

## Supplementary Material

Supplementary Dataset 1

## Figures and Tables

**Table 1:** Metagenomic sequencing coverage and the alpha diversity.

Sample	High quality reads^#^ (metagenomic data)	Assembled Contig^#^	Average N50 length	Annotated Genes^#^	Average Length
Buffalo (*n* = 10)	63456244	171549	4705	304800	594
SEM (Buffalo)	3236739	6325	835	100733	63
Cattle (*n* = 10)	60219098	100122	8182	191639	639
SEM (Cattle)	1579564	6146	553	83993	79
**Sample**	**OTU^#^ (16S rRNA sequencing data)**	**Observed Species**	**Chao 1 Index**	**Shannon Index**	**Simpson Index**
Buffalo (*n* = 10)	2021	2003.19	4011.50	9.85	0.9973
SEM (Buffalo)	370	365.68	1065.66	0.40	0.0010
Cattle (*n* = 10)	2309	2294.62	4530.06	9.63	0.9952
SEM (Yak)	579	173.88	847.99	0.82	0.0039

**Table 2:** Significantly different intestinal genera between cattle and buffalo.

Genus	Relative contribution (%)		Median, range (%)		Enriched	Adjusted *P*-value
	Cattle	Buffalo	Cattle	Buffalo		
*Streptococcus*	0.010	0.000	0.008 (0–0.038)	0 (0–0)	Cattle	0.006
*Pseudobutyrivibrio*	0.010	0.000	0.006 (0–0.038)	0 (0–0)	Cattle	0.015
*Anaerorhabdus*	0.044	0.010	0.038 (0–0.121)	0 (0–0.036)	Cattle	0.021
*Campylobacter*	0.888	0.000	0 (0–7.328)	0 (0–0)	Cattle	0.035
*Blautia*	0.014	0.005	0.011 (0–0.029)	0 (0–0.033)	Cattle	0.024
*Sutterella*	0.000	0.025	0 (0–0)	0.018 (0–0.085)	Buffalo	0.002
*Coprococcus*	0.125	0.035	0.122 (0.025–0.331)	0.012 (0–0.147)	Buffalo	0.005
*Parasutterella*	0.019	0.087	0.015 (0–0.057)	0.061 (0–0.205)	Buffalo	0.013
*Paludibacter*	0.147	0.464	0.044 (0–0.742)	0.374 (0.075–1.615)	Buffalo	0.026
*Dorea*	0.058	0.127	0.036 (0–0.154)	0.122 (0.066–0.238)	Buffalo	0.015

Note: Adjusted *P* values (*P* < 0.05) for the Wilcoxon rank-sum test are listed.

**Table 3:** The significantly different pathways of intestinal microbiota between cattle and buffalo.

Pathway ID	Reporter score (>0, enriched in buffalo; <0, in cattle)	Number of all KOs in the pathway	Number of detected KOs in the pathway (coverage %)	Pathway annotation	Level 1-Pathway
Map01230	5.773	226	194 (85%)	Biosynthesis of amino acids	Metabolism
Map02040	3.245	37	37 (100%)	Flagellar assembly	Cellular Processes
Map00770	3.221	36	32 (89%)	Pantothenate and CoA biosynthesis	Metabolism
Map00540	3.147	38	36 (95%)	Lipopolysaccharide biosynthesis	Metabolism
Map02020	2.969	428	323 (75%)	Two-component system	Environmental Information
Map02060	2.592	79	67 (85%)	Phosphotransferase system (PTS)	Environmental Information
Map00550	2.522	40	35 (88%)	Peptidoglycan biosynthesis	Metabolism
Map00290	2.517	17	16 (94%)	Valine, leucine and isoleucine biosynthesis	Metabolism
Map00780	2.357	19	17 (89%)	Biotin metabolism	Metabolism
Map00020	2.284	56	46 (82%)	Citrate cycle (TCA cycle)	Metabolism
Map00531	2.062	15	14 (93%)	Glycosaminoglycan degradation	Metabolism
Map00520	2.060	144	113 (78%)	Amino sugar and nucleotide sugar metabolism	Metabolism
Map00500	1.934	101	78 (77%)	Starch and sucrose metabolism	Metabolism
Map00450	1.884	28	25 (89%)	Selenocompound metabolism	Metabolism
Map00511	1.884	18	16 (89%)	Other glycan degradation	Metabolism
Map00030	1.874	75	59 (79%)	Pentose phosphate pathway	Metabolism
Map02030	1.794	26	25 (96%)	Bacterial chemotaxis	Cellular Processes
Map00620	−3.530	90	77 (86%)	Pyruvate metabolism	Metabolism
Map00010	−3.337	93	76 (82%)	Glycolysis/Gluconeogenesis	Metabolism
Map03010	−2.948	143	109 (76%)	Ribosome	Genetic Information
Map00633	−2.674	17	17 (100%)	Nitrotoluene degradation	Metabolism
Map00710	−1.971	36	29 (81%)	Carbon fixation in photosynthetic organisms	Metabolism

Note: A reporter score of >1.6 (90% confidence according to normal distribution) could be used as a detection threshold for significantly different pathways.

**Table 4:** Milk components of cattle and buffalo.

Species	Protein (g/100 g)	Fat (g/100 g)	Lactose (g/100 g)	Total solids (g/100 g)
Buffalo (*n* = 10)	5.96 ± 0.5^a^	7.16 ± 0.52^a^	4.08 ± 0.76	16.35 ± 1.42^a^
Cattle (*n* = 10)	4.15 ± 0.29	4.98 ± 0.58	4.96 ± 0.36*	13.67 ± 1.82
**Species**	**Na (mg/100 g)**	**Mg (mg/100 g)**	**K (mg/100 g)**	**Ca (mg/100 g)**
Buffalo (*n* = 10)	38.92 ± 1.88	18.21 ± 0.78^a^	91.82 ± 17.89	182.53 ± 13.36^a^
Cattle (*n* = 10)	33.09 ± 1.15	11.25 ± 1.21	136.16 ± 24.46*	104.69 ± 6.92

Note: ^a^represents significantly different milk components.
